# Editorial: Dual Role of Microglia in Health and Disease: Pushing the Balance Towards Repair

**DOI:** 10.3389/fncel.2020.00259

**Published:** 2020-08-28

**Authors:** Liliana Bernardino, Cinzia Volonté, Maria Beatrice Passani, Raquel Ferreira

**Affiliations:** ^1^Faculty of Health Sciences, Health Sciences Research Centre (CICS-UBI), University of Beira Interior, Covilhã, Portugal; ^2^CNR, Antonio Ruberti Institute for Systems Analysis and Computer Science (IASI), Rome, Italy; ^3^Fondazione Santa Lucia IRCCS, Rome, Italy; ^4^Section of Clinical Pharmacology and Oncology, Department of Health Sciences, University of Florence, Florence, Italy

**Keywords:** microglia, neuroinflammation, intercellular communication, neurodegenerative diseases, cytokines, phagocytosis

Microglial cells are innate immune cells in the Central Nervous System (CNS) that provide support and facilitate tissue repair through the avid production of several inflammatory mediators (e.g., chemokines and cytokines) and growth factors (e.g., BDNF). They also remove menacing foreign or pathogenic elements and ensure the establishment and maintenance of proper synaptic connections. These diverse functions are addressed by the contributors of the present Research Topic.

He et al. explored the relationship between microglia activation and chemokine expression in a context of neuropathic hypersensitivity. In their study, the tetracycline antibiotic minocycline was administered in the hippocampus after nerve injury and clearly inhibited allodynia (i.e., a condition in which pain is caused by an innocuous stimulus). This analgesic effect was produced at the molecular level via downregulation of toll-like receptors and suppression of chemokine expression, which further emphasizes the role of minocycline on microglia activity. Burmeister and Marriott reviewed the role of the glial-derived interleukin (IL)-10 family of cytokines, which includes IL-19, IL-20, IL-22, and IL-24, in neuroinflammation. While IL-10 is generally accepted as a classical anti-inflammatory cytokine, IL-20 appears to have a pro-inflammatory effect and IL-22 can act either way. This reinforces the notion that our knowledge on these cytokines remains limited and warrants further attention to explore their potential in inflammatory brain disorders.

Blood coagulation factor fibrinogen has been associated to the development of depressive symptoms in Alzheimer's disease. In the context of brain dementia, Piers et al. showed how fibrinogen triggers an inflammatory microglial phenotype, assessed by genetic microarray analysis (microglial cell line) and proteome cytokine profiling (primary microglia). The consequent endoplasmic reticulum stress was blocked by inhibiting tumor necrosis factor (TNF)-α transcription or neuronal caspase activity, paving the way for the use of TNF inhibitors in inflammatory-mediated dementias. On a broader subject, Lee and Suk reviewed evidence on the role of protein kinases in neurodegenerative diseases such as Alzheimer's disease, Parkinson's disease, and amyotrophic lateral sclerosis. Specifically, authors review microglial activity via protein kinase modulation in inflammatory contexts to treat or alleviate symptoms manifested in neurodegenerative conditions. In amyotrophic lateral sclerosis, Vaz et al. evaluated the effects of wild-type and mutant superoxide dismutase 1 (SOD1) expression in microglia upon overexpression of wild-type (hSOD1WT) and mutated G93A (hSOD1G93A) protein, to uncover possible therapeutic targets. Both transduced cells displayed lower expression of anti-inflammatory targets but only microglia transduced with hSOD1G93A had higher expression of pro-inflammatory markers. Moreover, their exosomes were enriched with SOD1 and HMGB1. While overexpressed hSOD1WT attenuated activation, hSOD1G93A produced a reactive phenotype albeit with low responsiveness. To extend their previous works, two molecules known to elicit neuroregeneration, glycoursodeoxycholic acid and dipeptidyl vinyl sulfone, were successfully tested. Both exerted immunomodulatory properties over the hSOD1G93A microglia, but through specific molecular pathways.

In addition to exosomes, there are other types of vesicles that mediate extracellular communication. Grimaldi et al. investigated the therapeutic potential of microvesicles derived from microglial cells in a glioma context. Vesicles were first obtained from microglial cells stimulated with lipopolysaccharide and interferon-γ, and then infused into mice injected with glioma cells in their striatal brain region. The group observed the induction of a protective phenotype and reduced tumor size, which was attributed to the microvesicle cargo enriched in transcripts for several inflammation-related genes.

Regarding microglia-secreted hormones, the potential of insulin-like growth factor-1 (IGF-1) was evidenced by several articles. Myhre et al. researched and compared IGF-1 and TNF expression, and the amyloid-β (Aβ) plaque load in adult and old APPswe/PS1dE9 transgenic vs. wild-type mice. In the wild-type animals, IGF-1 remained constant whereas TNF expression increased with aging. IGF-1 and TNF levels were found elevated in the aged transgenic group; Aβ plaque load increased and plateaued in this group. The main contributor of IGF-1 was microglial cells and the ability to retain expression alludes to the therapeutic potential of modulating this cell population in Alzheimer's disease. IGF-1 is also pivotal for neurotransmission, particularly in the developing and mature auditory system. Fuentes-Santamaría et al. used knockout-out mice to study the mechanisms involved in excitatory synaptic plasticity occurring in the cochlear nuclei. The *Igf1*^−/−^ mice exhibited lower levels in markers related to microglial and astrocyte activation, higher glutamate transporter 1 expression and reduced metabotropic glutamate receptor expression. Therefore, the imbalances in neurotransmission observed in cochlear nuclei may be a consequence of microglia and astrocyte impairment. Wlodarczyk et al. showed that the stimulation of the colony stimulating factor-1 receptor (CSF1R) promoted the expansion of CD11c-positive microglia. This cell subpopulation provided primary myelination and constituted a major source of IGF-1, which is critical for neuronal survival. Using a model of experimental autoimmune encephalomyelitis, which mimics human inflammatory demyelinating disease, the CSF1R stimulation caused CCL2 overexpression and an increase of CD11c-positive microglia, later reflecting the amelioration of symptoms and reduced demyelination.

Phagocytosis of cell debris and invading pathogens is also a hallmark of microglia activity. In this sense, Reichert and Rotshenker demonstrated how galectin-3 could trigger complement receptor 3-mediated phagocytosis of myelin debris. Galectin-3 knockdown reduced phagocytosis and changed cell morphology from “amoeboid-like” to “branched-like” microglia. This effect was produced by reorganization of actin filaments and cofilin inactivation. Galactin-3 acts by activating cofilin and then K-Ras signaling, thus controlling microglia cytoskeleton and phagocytic activity. In addition, Akhmetzyanova et al. assessed if microglia phagocytic activity in the acute period of spinal cord injury could influence post-traumatic processes by transplanting genetically modified microglial cells (Ad5-GDNF or Ad5-EGFP) into the spinal injured area. While Ad5-GDNF microglial have decreased phagocytic activity, Ad5-EGFP microglia are amoeboid active phagocytic cells. The area of intact nervous tissue was lower in the first group but differences in functional recovery were not observed. Hence, further studies are warranted to determine how enhanced tissue sparing can positively correlate with a functional outcome.

Finally, Lively et al. evaluated the importance of sex differences and age in microglial responses. Most animal studies favor the use of male adult rodents. Hence, microglia from sex-segregated postnatal day 1 and postnatal day 21 rats were analyzed regarding their response to pro- or anti-inflammatory stimulation. The extensive evaluation of migration ability, expression of inflammatory mediators and phagocytosis-related molecules, revealed several alterations at the transcriptional and functional level associated to age but not to sexual dimorphism.

Altogether, we expect the work presented in this topic will shed light on the role of microglia on intercellular communication in the brain parenchyma, thus stimulating a further scientific debate and encouraging novel therapeutic approaches to efficiently repair the injured CNS.

## Author Contributions

All authors listed have made a substantial, direct and intellectual contribution to the work, and approved it for publication.

## Conflict of Interest

The authors declare that the research was conducted in the absence of any commercial or financial relationships that could be construed as a potential conflict of interest.

